# Comparative Effectiveness of High-Intensity Interval Training and Moderate-Intensity Continuous Training for Cardiometabolic Risk Factors and Cardiorespiratory Fitness in Childhood Obesity: A Meta-Analysis of Randomized Controlled Trials

**DOI:** 10.3389/fphys.2020.00214

**Published:** 2020-04-03

**Authors:** Jingxin Liu, Lin Zhu, Yu Su

**Affiliations:** ^1^Research Center for Physical Fitness and Health Promotion of Adolescent, Guangzhou Sport University, Guangzhou, China; ^2^College of Physical Education, Shaoguan University, Shaoguan, China

**Keywords:** high-intensity interval training, pediatric obesity, weight loss, cardiorespiratory fitness, lipid metabolism

## Abstract

**Purpose:** The main objective of this meta-analysis was to compare the effectiveness of high-intensity interval training (HIIT) and of moderate-intensity continuous training (MICT) on cardiometabolic health in childhood obesity and determine whether HIIT is a superior form of training in managing obese children's metabolic health.

**Methods:** Relevant studies published in PubMed, Web of Science, Embase, the Cochrane Library, EBSCO, and CNKI were searched, restricted to those published from inception to 1 October 2019. Only randomized controlled trials (RCTs) depicting the effect of HIIT on childhood obesity were included.

**Results:** Nine RCTs involving 309 participants were included in the meta-analysis. Among the 309 participants, 158 subjects were randomized for HIIT, while the others were randomized for MICT. Significant differences were observed in the body weight (mean difference [MD] = −5.45 kg, *p* = 0.001), body mass index (BMI; MD = −1.661 kg/m^2^, *p* = 0.0001), systolic blood pressure (SBP; MD = −3.994 mmHg, *p* = 0.003), and diastolic blood pressure (DBP; MD = −3.087 mmHg, *p* = 0.0001) in the HIIT group relative to the baseline values. Similar effects were found in the MICT group, as depicted by the significantly decreased values for body weight (MD = −4.604 kg, *p* = 0.0001), BMI (MD = −2.366 kg/m^2^, p = 0.0001), SBP (MD = −3.089 mmHg, *p* = 0.019), and DBP (MD = −3.087 mmHg, *p* = 0.0001). However, no significant differences were observed in the changes in body weight, BMI, SBP, or DBP between the HIIT and MICT groups. Furthermore, our studies showed that both HIIT and MICT could significantly improve VO_2peak_ (HIIT, MD = 4.17 ml/kg/min, 95% CI: 3.191 to 5.163, *p* = 0.0001; MICT, MD = 1.704 ml/kg/min, 95% CI: 0.279 to 3.130, *p* = 0.019). HIIT also showed more positive effects on VO_2peak_ (SMD = 0.468, 95% CI: 0.040 to 0.897, *p* = 0.006) than MICT.

**Conclusion:** HIIT positively affects the cardiometabolic risk factors in childhood obesity. Similar positive effects on body composition and blood pressure were established. Moreover, HIIT can improve cardiorespiratory fitness more significantly than MICT. These findings indicate that HIIT may be an alternative and effective training method for managing childhood obesity.

**PROSPERO Registration Number:** CRD42018111308.

## Introduction

Childhood obesity, defined by the World Health Organization (WHO) as abnormal or excessive fat accumulation that can eventually pose health risks, is one of the most serious global public health challenges of the twenty first century. Childhood obesity is highly prevalent; the latest epidemiological studies demonstrated that 107.7 million children worldwide were obese in 2015 and that the growth rate of childhood obesity was greater than that of adult obesity (Afshin et al., 2017). Strong evidence indicates that excess weight during childhood is a predictor of future obesity and can increase cardiometabolic risks, such as insulin resistance, dyslipidemia, hypertension, and poor cardiorespiratory fitness, in obese children (Gepstein and Weiss, 2019; Wibaek et al., 2019). Emerging evidence also shows that cardiorespiratory fitness, as an important predictor of cardiovascular disease, not only helps prevent cardiovascular disease (Castro-Pinero et al., 2017; Kachur et al., 2017; Lavie et al., 2019) but also plays a regulatory role in reducing the risk of obesity in children (Lahoz-Garcia et al., 2018; Yu et al., 2018; Prieto-Benavides et al., 2019). Therefore, adverse changes in the aforementioned contributing factors will inevitably increase the risk of cardiometabolic diseases, such as type 2 diabetes and cardiovascular disease, in adulthood (Juonala et al., 2011; Chung et al., 2018).

Exercise is a critical component of childhood obesity management because it can improve body composition and maintain cardiometabolic health. Both the American College of Sports Medicine and the WHO have strongly recommended that children allocate at least 60 min per day to moderate to vigorous physical activities and engage in high-intensity exercises at least three times per week. Moderate-intensity continuous training (MICT) is the traditional method of increasing physical activity. It is an effective way of reducing body fat and cardiometabolic risk in obese children. However, the effectiveness of MICT relies on long-duration sessions (Alberga et al., 2013; Sigal et al., 2014), and only a few children can achieve the required effective duration (Fan and Cao, 2017). Therefore, other time-efficient exercise modalities for obese children and adolescents should be explored.

High-intensity interval training (HIIT), defined as alternating short bursts of high-intensity exercise and light exercise or passive recovery periods, has been considered a good alternative, more time-efficient strategy to MICT. Existing systematic reviews and meta-analyses have revealed that HIIT has more significant effects on abdominal and visceral fat reduction and cardiorespiratory fitness improvement in overweight and obese adults than MICT (Maillard et al., 2018; Roy et al., 2018). Moreover, HIIT can reduce metabolic risk factors in type 2 diabetes more effectively than MICT (Costigan et al., 2015; Hannan et al., 2018). Wewege et al. (2017) showed that HIIT can save 40% of the time committed to MICT, with similar magnitude changes in body fat and waist circumference (WC).

HIIT studies have focused more on adults and patients with chronic disease than on obese children and adolescents. Moreover, there is no consensus or indication as to whether HIIT is superior or a good alternative training modality to MICT for reducing the cardiometabolic risk factors in childhood obesity. The purpose of our meta-analysis was to compare the effectiveness of HIIT and MICT in reducing the abovementioned cardiometabolic risk factors and determine which HIIT modality is effective and time-efficient in managing the abovementioned risks.

## Methods

The meta-analysis protocol was registered with the International Prospective Register of Systematic Reviews (CRD42018111308), and the study was conducted according to the recommendations of the Preferred Reporting Items for Systematic Review and Meta-Analysis (PRISMA) protocols (Shamseer et al., 2015). The details of the meta-analysis protocol have been published previously by Liu et al. (2019a).

### Search Strategy

Relevant studies published in PubMed, Web of Science, Embase, the Cochrane Library, EBSCO, and CNKI were searched, restricted to those published from inception to 1 December 2019. A systematic literature search strategy was employed using the patient/problem, intervention, control/comparison, outcome, study design principle. The search strategy is detailed in Supplementary Appendix e-1. Moreover, we screened the list of included articles cited in the relevant journals and references to identify other potentially eligible studies.

### Inclusion Criteria

The studies were regarded eligible for inclusion if they met the following criteria. (1) Participants: The participants were 8- to 16-year-old children and adolescents diagnosed with childhood obesity. Childhood obesity in the present research was defined on the basis of a body mass index (BMI) ≥95th percentile for the age and gender subgroups (see Centers for Disease Control and Prevention in Kuczmarski et al., 2002) and age- and gender-specific cutoff points (males, >21.6 kg/m^2^; females, >21.57 kg/m^2^; see International Obesity Task Force in Cole et al., 2000) and a BMI standard deviation score (BMI-SDS) of >2 (see WHO in de Onis et al., 2007). (2) Intervention: The participants only received HIIT interventions, and HIIT was compared with MICT. We excluded the combination of HIIT or MICT with other types of exercises. HIIT intensity was defined as maintaining 80–100% of the peak heart rate (HR_peak_) or VO_2peak_ (Keating et al., 2017) for 30 s to 4 min, interspersed to a maximum of 4 min of passive recovery or low-intensity aerobic exercise. Exercise intensity, prescribed as a percentage of heart rate (HR) reserve, maximal aerobic speed, and rate of perceived exertion equivalent to 80–100% of HR_peak_ and VO_2peak_, was included in HIIT (Garber et al., 2011). MICT intensity was defined as maintaining 40–79% of HR_peak_ or VO_2peak_ for 20–60 min. The HIIT and MICT interventions entailed the same training frequencies and durations. (3) Outcomes: The studies reported at least one of the following data types related to the cardiometabolic risk factors: body composition (e.g., body weight, BMI, WC, and body fat percentage), glucose metabolism (e.g., blood fasting glucose, blood fasting insulin, and homeostatic model assessment of insulin resistance [HOMA-IR]), blood pressure (e.g., systolic blood pressure [SBP] and diastolic blood pressure (DBP), lipid metabolism (e.g., high-density lipoprotein cholesterol [HDL-c], low-density lipoprotein cholesterol [LDL-c], triglyceride [TG], and total cholesterol [TC]), and cardiorespiratory fitness (e.g., VO_2peak_). (4) Design: Only randomized controlled trials (RCTs) were included.

### Selection of Studies and Data Extraction

Two independent reviewers performed a study screening process following the PRISMA guidelines. A bibliographic reference manager (EndNote X7, Thomson Reuters) was used to remove duplicate entries. After screening the titles and abstracts, the studies that did not meet our eligibility criteria were excluded. The remaining studies were evaluated by reading their full texts and making a final decision. All differences between the reviewers' viewpoints were resolved through discussions or consultation with a third reviewer.

The data were extracted from each study following the predesigned guideline on unified standardization by two independent reviewers. The following data were extracted: first author's name, publication year, country, participant characteristics (gender and age), number of participants, intervention protocols (training intensity and time, interval intensity and time, and frequency and duration), main outcomes, and dropout rates. If duplicate data were observed in the different studies during data collection, then additional comprehensive studies were extracted, and the authors were consulted if data were missing.

### Risk of Bias Assessment

The methodological quality of the included studies was evaluated by two independent authors (JL and LZ) who used Cochrane Collaboration's tools to check for random sequence generation, allocation concealment, blinding, incomplete outcome data, selective reporting, and other biases; the evaluation results were categorized into high-risk, low-risk, and unclear grades (Higgins et al., 2011).

### Data Analysis and Synthesis

Data analysis was performed using Review Manager 5.3.5 (The Nordic Cochrane Centre, The Cochrane Collaboration, Copenhagen, Denmark) and Stata 12.0. The mean difference (MD) with a 95% confidence interval (95% CI) was calculated for the effect size either between HIIT and MICT or between pre-intervention and post-intervention in each group. The standard mean difference (SMD) with 95% CI was selected due to the varying units or the large differences among the studies. The change in values from the baseline in each group was calculated with the formula *M* = | *M*_1_−*M*_2_ |, where *M* is the effect mean, *M*_1_ is the effect mean of the baseline, and *M*_2_ is the end value mean, followed by the formula $S^{2}=S_{1}^{2}+S_{2}^{2}-2\times R\times S_{1}\times S_{2}$, where *S* is the standard deviation of the effect, *S*_1_ is the standard deviation of the baseline value, *S*_2_ is the final standard deviation, and *R* is a constant (0.4 or 0.5). *I*^2^ statistic and *Q* statistic were used to estimate the heterogeneity between two studies.

Values of 0% ≤ *I*^2^ < 25% indicate trivial heterogeneity, 25% ≤ *I*^2^ < 50% indicate small heterogeneity, 50% ≤ *I*^2^ < 75% indicate moderate heterogeneity, and 75% ≤ *I*^2^ < 100% indicate high heterogeneity. If moderate or high heterogeneity exists between the studies, then the random effect model is used; otherwise, the fixed-effect model is adopted. If moderate or high heterogeneity exists between the studies, then sensitivity analysis and subgroup analysis are conducted. Here, sensitivity analysis was performed by changing the pooled model or by adopting a 1 × 1 exclusion approach.

Subgroup analysis was performed to examine whether the training parameters in the included studies positively affect the cardiometabolic risk factors. The following intervention features were examined: training session time, total training duration, and type of interval. The subgroup analysis of each outcome was carried out by referring to at least two studies, and a chi-square test was conducted to assess heterogeneity between subgroups. Egger's test was carried out to assess publication bias. Univariate meta-regression analyses were not conducted due to the limited number of studies.

## Results

### Study Selection

**Figure 1:**
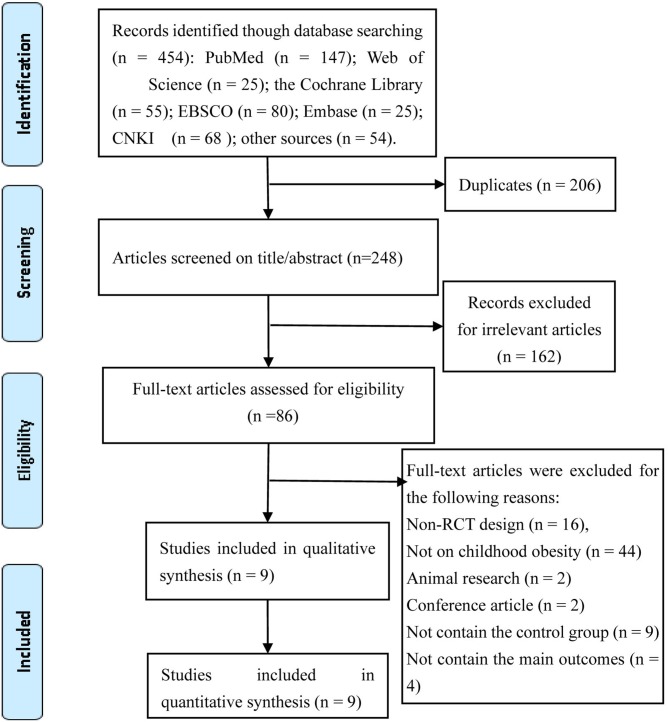
Selection process of eligible studies.

A total of 454 studies were identified in accordance with our search strategy. Among the 454 studies, nine RCTs were considered eligible after the duplicates were removed, the titles/abstracts were screened, and the full texts were reviewed. The selection process of identifying eligible studies is shown in Figure 1.

### Study Characteristics and Participants

The primary characteristics of the included studies are shown in Table 1. A total of 309 participants were included in the meta-analysis, of whom 166 participants were males and the remaining were females. The gender composition of the participants in the HIIT group was 85 males and 73 females, while that of the MICT group was 81 males and 73 females. The mean age of the participants was 13.31 ± 1.94 years old, and the mean BMI was 31.89 ± 3.09 kg/m^2^.

**Table 1:** Characteristics of the included studies.

**References**	**Country**	**Study design**	**Main characteristics of the subjects**	**Interventions**		**Outcomes**	**Drop-out subjects**
				**HIIT**	**MICT**		
Morrissey et al. (2018)	France	RCT	HIIT: mean age was 15.0 ± 1.4 years, mean BMI was 35.0 ± 3.0 kg/m^2^, four males and 12 females, *n* = 16 MICT: mean age was 15.0 ± 1.6 years, mean BMI was 34.0 ± 1.0 kg/m^2^, four males and nine females, *n* = 13	Training at 90–95% HR_max_ for 120–150 s, with a recovery interval at 55% HR_max_ for 90 s. 4–6 bouts per session, three times a week for 12 week	Training at 60–70% HR_max_, 40–60 min per session, 3 times a week for 12 weeks	BW; BMI; BF (%); SBP; DBP; FG; FI; HOMA-IR; TG; TC	0
Dias et al. (2018)	Norway and Australia	RCT	HIIT: mean age was 12.4 ± 1.9 years, 16 males and 17 females, *n* = 33 MICT: mean age was 11.9 ± 2.4 years, 15 males and 17 females, *n* = 32.	4*4 min bouts at 85–95% HR_max_, with an active recovery interval at 50–70% HR_max_ for 3 min, three times a week for 12 weeks	Training at 60–70% HR_max_ for 44 min, three times a week for 12 weeks	BW; BMI; BF (%); HDL-c; IR; FG; TG; LDL-c; TC; VO_2peak_	24
Lazzer et al. (2017)	Italy	RCT	HIIT: mean age was 16.8 ± 0.7 years, 10 males, *n* =10 MICT: mean age was 16.1 ± 1.1 years, nine males, *n* = 9	Training at 100% VO_2peak_ for 40 s, interspersed with 5 min of walking at 40% VO_2peak_, 37 min/session, two sessions per day for 3 weeks	Training at 70% VO_2peak_ for 30 min, 31 min per session, two sessions per day for 3 weeks	BW; BMI; VO_2peak_	0
Mahgoub and Aly (2015)	Egypt	RCT	HIIT: mean age was 13.66 ± 1.11 years, mean BMI was 30.42 ± 1.58 kg/m^2^, six males and nine females, *n* = 15 MICT: mean age was 13.73 ± 1.03 years, five males and 10 females, mean BMI was 30.18 ± 1.67 kg/m^2^, *n* = 15	Training at 80% VO_2peak_ for 2 min, with 1 min rest intervals, 30 min per session, 8 weeks Note: 75% VO_2peak_ in the first 4 weeks	Training at 50–60% VO_2peak_ for 30 min for 8 weeks	TC; TG; LDL-c; HDL-c	0
Starkoff et al. (2014)	USA	RCT	HIIT: mean age was 14.9 ± 1.6 years, mean BMI was 36.5 ± 5.4 kg/m^2^, eight males and 10 females, *n* = 18 MICT: mean age was 14.5 ± 1.4 years, mean BMI was 38.7 ± 6.7 kg/m^2^, six males and 10 females, *n* = 16	Training at 90–95% HR_max_ for 2 min, with an active recovery interval at 55% HR_max_ for 1 min, 10 bouts per session, 3 times a week for 6 weeks	Training at 65–70% HR_max_ for 30 min, three times a week for 6 weeks	VO_2peak_	7
Xiuming (2014)	China	RCT	HIIT: mean age was 10.20 ± 0.45 years, mean BMI was 28.0 ± 1.19 kg/m^2^, 20 males and 10 females, n = 30 MICT: mean age was 10.40 ± 1.34 years, mean BMI was 28.50 ± 1.11 kg/m^2^, 22 males and eight females, *n* = 30	Training 90–95% HR_max_ for 1 min, and then gradually to 50% HR_max_ within 1, 30 min per session, twice a week for 12 weeks	Training at 80% HR_max_ for 30–60 min, twice a week for 12 weeks	BW; BMI; SBP; FG; DBP; FI; TC; HDL-c; LDL-c; TG; HOMA-IR	0
Murphy et al. (2015)	USA	RCT	HIIT: mean age was 13.7 ± 2.0 years, two males and five female, *n* = 7 MICT: mean age was 14.3 ± 1.2 years, one male and five females, *n* = 6	Training at 80–90% HR_max_ for 1 min, with an active interval at 60% HR_max_ for 2, 30 min per session, three times a week for 4 weeks	Training at 65% HR_max_ for 30 min, three times a week for 4 weeks.	BW; BMI; BF (%); SBP; SDP; VO_2peak_	0
Koubaa et al. (2013)	Tunisia	RCT	HIIT: mean age was 13 ± 0.8 years, mean BMI was 30.2 ± 3.6 kg/m^2^, 14 males, *n* = 14 MICT: mean age was 12.9 ± 0.5 years, mean BMI was 30.8 ± 2.9 kg/m^2^, 15 males, *n* = 15	Training at 80% VO_2max_ for 2 min, interspersed with 1 min recovery, three times per week for 12 weeks	Training at 60–70% VO_2max_ for 30–40 min, three times per week for 12 weeks	BW; BMI; TC; HDL-c; LDL-c; SBP; WC; DBP; TG; VO_2peak_	1
Corte de Araujo et al. (2012)	Brazil	RCT	HIIT: mean age was 10.7 ± 0.7 years, mean BMI was 30.8 ± 3.7 kg/m^2^, five males and 10 females, *n* = 15 MICT: mean age was 10.4 ± 0.9 years, mean BMI was 29.6 ± 4.0 kg/m^2^, four males and 11 females, *n* = 15	Training at 100% peak velocity for 1 min, with a 3 min interval at 50% peak velocity, twice a week for 12 weeks	Training at 80% HR_max_ for 30–60 min, twice a week for 12 weeks	BW; BMI; WC; SBP; DBP; FG; FI; HDL-c; LDL-c; HOMA- IR; TG; TC	0

*BW, body weight; BMI, body mass index; WC, waist circumference; SBP, systolic blood pressure; DBP, diastolic blood pressure; FG, fasting glucose; FI, fasting insulin; TG, triglyceride; TC, total cholesterol; HDL-c, high-density lipoprotein cholesterol; LDL-c, low-density lipoprotein cholesterol; RCT, randomized controlled trial; HIIT, high-intensity interval training; MICT, moderate-intensity interval training*.

Most of the studies monitored training intensity with the use of HR monitors or monitored oxygen consumption to ensure adequate training intensity. The nine studies applied HIIT strategies involving a variety of intensities and interval durations; HIIT strategies could be divided into short-interval training programs and long-interval training programs. The short-interval training programs consisted of training performed at intensities >90% VO_2peak_ or HR_max_ for 30 s to 2 min intervals with 30 s to 1 min recovery (Corte de Araujo et al., 2012; Xiuming, 2014; Murphy et al., 2015; Lazzer et al., 2017). The long-interval training programs consisted of training performed at intensities of 80% VO_2peak_ or 85–100% HR_max_ fort 2–4 min intervals with 1–3 min recovery (Koubaa et al., 2013; Starkoff et al., 2014; Mahgoub and Aly, 2015; Dias et al., 2018; Morrissey et al., 2018). The majority of the MICT programs consisted of training performed at 60–80% HR_max_ or 60–70% VO_2peak_ for 30–40 min. The frequency range of HIIT was two to three times per week; most of the reviewed studies mentioned three times per week. The duration of HIIT usually lasted for 3–12 weeks; most of the reviewed studies mentioned 12 weeks.

Among the included studies, only Corte de Araujo et al. (2012); Dias et al. (2018), and Lazzer et al. (2017) reported methods to assess dietary intake or energy intake. No adverse events were mentioned in any of the studies.

### Methodological Quality of Included Studies

**Figure 2:**
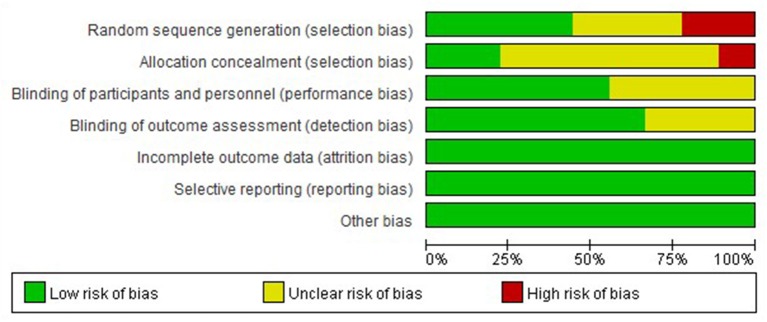
Review judgment of risk bias for each item: percentages across all included studies.

**Figure 3:**
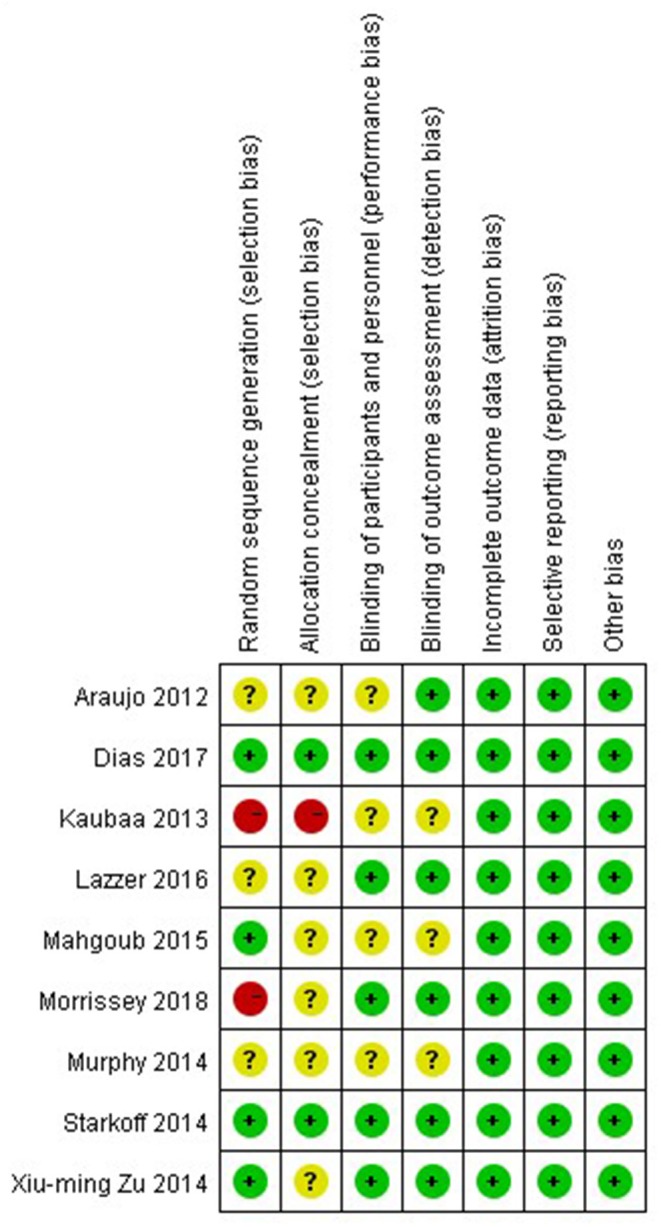
Summary of risk bias: review authors' judgment of risk bias for each item and adolescent.

The nine studies were assessed for risk bias (Figures 2, 3). Among the included studies, four studies cited random sequence generation, two studies mentioned concealment, five studies involved the blind participation of respondents and personnel, and six studies described the blind participation of outcome evaluators. None of the included studies reported incomplete outcome data, selective reports bias, or other biases.

### Meta-Analysis

#### Body Composition

**Figure 4:**
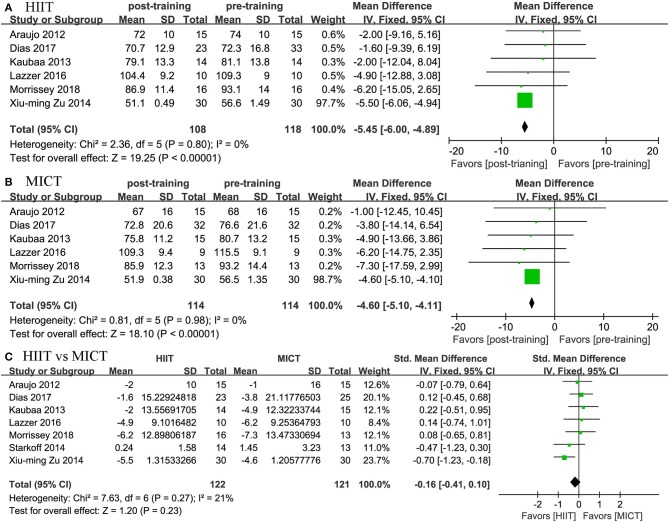
Forest plot for changes in body weight. **(A)** Within-group effects of HIIT. **(B)** Within-group effects of MICT. **(C)** Between-group effects of HIIT and MICT.

**Figure 5:**
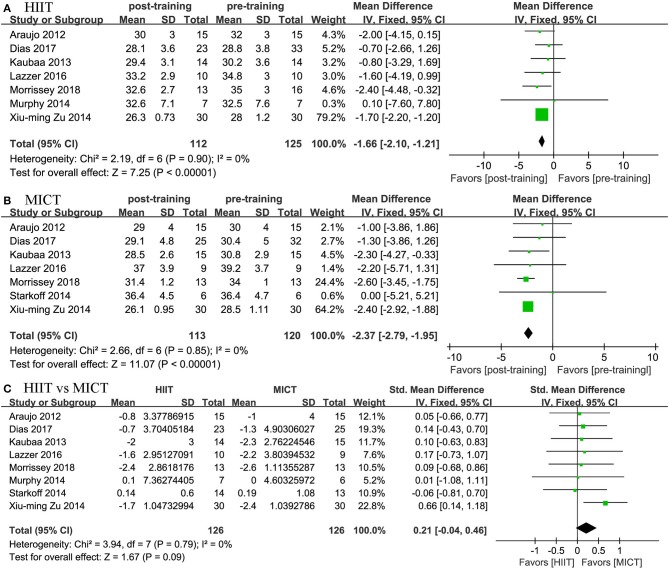
Forest plot for changes in BMI. **(A)** Within-group effects of HIIT. **(B)** Within-group effects of MICT. **(C)** Between-group effects of HIIT and MICT.

Eight studies assessed the effects of HIIT and MICT on body composition, as measured by body weight (*n* = 7), BMI (*n* = 8), WC (*n* = 3), and body fat percentage (*n* = 5). Significant differences were observed for body weight (MD = −5.45 kg, 95% CI: −6.003 to −4.894, *p* = 0.001) and BMI (MD = −1.661 kg/m^2^, 95% CI: −2.109 to −1.213, *p* = 0.0001) in the HIIT group (Figures 4, 5) relative to the baseline values. Similar effects were found in the MICT group (Figures 4, 5), as indicated by the significantly decreased body weight (MD = −4.604 kg, 95% CI: −5.103 to −4.106, *p* = 0.0001), BMI (MD = −2.366 kg/m^2^, 95% CI: −2.785 to −1.947, *p* = 0.0001), and WC (MD = −6.468 cm, 95% CI: −11.546 to −1.389, *p* = 0.013). However, no significant differences were observed in the changes in body weight (SMD = −0.16, 95% CI: −0.41 to 0.10, *p* = 0.23), BMI (SMD = 0.21, 95% CI: −0.04 to 0.46, *p* = 0.09), WC (MD = −0.342 cm, 95% CI: −3.204 to 2.520, *p* = 0.815), or body fat percentage (MD = −0.253%, 95% CI: −1.392 to 0.885, *p* = 0.663) between the HIIT and MICT interventions (Table 2).

**Table 2:** Summary of the meta-analysis.

**Outcomes**			**Within-group effects**		**HIIT vs. MICT**
			**HIIT**	**MICT**	
Body weight	N		6	6	7
	ES (95% CI)		MD: −5.45 (−6.003, −4.894)	MD: −4.604 (−5.103, −4.106)	SMD: −0.16 (−0.41, 0.10)
	Heterogeneity	*I*^2^	0	0	21%
		*P*	0.797	0.977	0.27
	*P*		0.0001	0.0001	0.23
BMI	N		7	7	8
	ES (95% CI)		MD: −1.661 (−2.109, −1.213)	MD: −2.366 (−2.785, −1.947)	SMD: 0.21 (−0.04, 0.46)
	Heterogeneity	*I*^2^	0	0	0
		*P*	0.851	0.851	0.79
	*P*		0.0001	0.0001	0.09
WC	N		2	2	3
	ES (95% CI)		MD: −4.575 (−9.506, 0.356)	MD: −6.468 (−11.546, −1.389)	MD: −0.342 (−3.204, 2.520)
	Heterogeneity	*I*^2^	2.4%	0	28.6%
		*P*	0.311	0.818	0.247
	*P*		0.069	0.013	0.815
Body fat (%)	N		4	4	5
	ES (95% CI)		MD: −0.792 (−2.551, 0.967)	MD: −0.156 (−1.985, 1.674)	MD: −0.253 (−1.392, 0.885)
	Heterogeneity	*I*^2^	12.4%	29.8%	0
		*P*	0.331	1.042	0.607
	*P*		0.378	0.867	0.663
VO_2peak_	N		4	4	4
	ES (95% CI)		MD: 4.17 (3.191, 5.163)	MD: 1.704 (0.279, 3.130)	MD:2.497 (1.151, 3.843)
	Heterogeneity	*I*^2^	0	0	0
		*P*	0.822	0.819	0.636
	*P*		0.0001	0.019	0.0001
SBP	N		4	4	4
	ES (95% CI)		MD: −3.994 (−6.942, −1.045)	MD: −3.089 (−5.679, −0.498)	MD: −1.208 (−2.603, 0.186)
	Heterogeneity	*I*^2^	78.4%	60%	47.2%
		*P*	0.003	0.058	0.128
	*P*		0.008	0.019	0.089
DBP	N		4	4	4
	ES (95% CI)		MD: −3.087 (−4.083, −2.092)	MD: −2.481 (−3.551, −1.410)	MD: 1.213 (−2.597, 5.023)
	Heterogeneity	*I*^2^	24.0%	23.1%	77.3%
		*P*	0.267	0.272	0.004
	*P*		0.0001	0.0001	0.533
TC	N		6	6	6
	ES (95% CI)		MD: −0.221 (−0.594, 0.108)	MD: −0.265 (−0.635, 0.124)	MD: −0.141 (−0.619, 0.337)
	Heterogeneity	*I*^2^	95.8%	97.2%	95.4
		*P*	0.0001	0.1963	0.0001
	*P*		0.1442	0.182	0.563
HDL–c	N		5	5	5
	ES (95% CI)		MD: 0.198 (−0.162, 0.557)	MD: 0.120 (−0.035, 0.275)	MD: 0.086 (−0.164, 0.337)
	Heterogeneity	*I*^2^	98.6%	92.9%	95.7
		*P*	0.0001	0.0277	0.0001
	*P*		0.281	0.130	0.499
LDL–c	N		5	5	5
	ES (95% CI)		MD: −0.495 (−1.059, 0.068)	SMD: −1.142 (−2.277, −0.007)	MD: −0.142 (−0.348, 0.063)
	Heterogeneity	*I*^2^	98.3%	91.9%	69.0%
		*P*	0.000	0.000	0.012
	*P*		0.3913	0.049	0.174
TG	N		6	6	6
	ES (95% CI)		MD: −0.085 (−0.271, 0.1)	MD: −0.048 (−0.110, 0.013)	MD: −0.052 (−0.113, 0.009)
	Heterogeneity	*I*^2^	89.6%	48.1%	20.7
		*P*	0.0001	0.086	0.278
	*P*		0.365	0.123	0.096
HOMA–IR	N		4	4	4
	ES (95% CI)		MD: −1.296 (−3.186, 0.595)	−0.814 (−2.187, 0.559)	SMD: −0.554 (−1.202, 0.093)
	Heterogeneity	*I*^2^	98.0%	95.9%	77.0%
		*P*	0.179	0.0001	0.005
	*P*		0.215	0.245	0.093
Fasting glucose	N		4	4	4
	ES (95% CI)		MD: −0.445 (−0.834, −0.056)	MD: 0.035 (−0.316, 0.387)	MD: −0.479 (−0.975, 0.017)
	Heterogeneity	*I*^2^	98.9%	91.2%	95.6%
		*P*	0.1508	0.001	0.0001
	*P*		0.025	0.843	0.059
Fasting insulin	N		3	3	3
	ES (95% CI)		SMD: −1.548 (−3.551, 0.454)	SMD: −0.343 (−711, 0.026)	SMD: −0.694 (−1.816, 0.428)
	Heterogeneity	*I*^2^	95.2%	0%	87.7%
		*P*	0.001	0.414	0.001
	*P*		0.130	0.068	0.225

#### Glucose Metabolism

Four studies assessed the effects of HIIT and MICT on glycemic control, as measured by fasting glucose (*n* = 4), fasting insulin (*n* = 3), and HOMA-IR (*n* = 3). The meta-analysis showed significantly reduced values for fasting glucose (MD = −0.445 mmol/L, 95% CI: −0.834 to −0.0.056, *p* = 0.025) in the HIIT group relative to the baseline values. The meta-analysis also showed that MICT had no significant effects based on the fasting glucose, fasting insulin, or HOMA-IR values. In the between-group comparison, the pooled results from the meta-analysis showed that HIIT elicited a higher change trend for fasting glucose (MD = −0.479 mmol/L), fasting insulin (SMD = −0.694), and HOMA-IR (SMD = −0.554); however, none of the changes in the effect values were significant (Table 2).

#### Lipid Metabolism

Six studies reported the effects of HIIT and MICT on blood lipids, as measured by TC (*n* = 6), HDL-c (*n* = 5), LDL-c (*n* = 5), and TG (*n* = 6). As the heterogeneity was large in all the comparisons (*I*^2^ = 95.8% for TC, *I*^2^ = 98.6% for HDL-c, *I*^2^ = 98.3% for LDL-c, and *I*^2^ = 89.6% for TG in HIIT vs. the baseline; *I*^2^ = 97.2% for TC, *I*^2^ = 92.9% for HDL-c, and *I*^2^ = 91.9% for LDL-c in MICT vs. the baseline), no evidence was derived from the pooled results for the effects of TC, HDL-c, LDL-c, or TG. MICT was determined to have no effects on TC, HDL-c, and TG relative to the baseline values. The change in HIIT was not significant; that is, no significant effects on the change in HIIT were found in any of the measures, unlike the change in MICT (Table 2).

#### Blood Pressure

**Figure 6:**
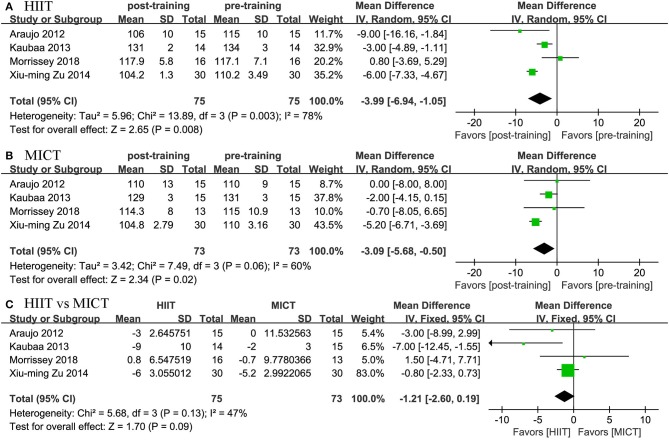
Forest plot for changes in SBP. **(A)** Within-group effects of HIIT. **(B)** Within-group effects of MICT. **(C)** Between-group effects of HIIT and MICT.

**Figure 7:**
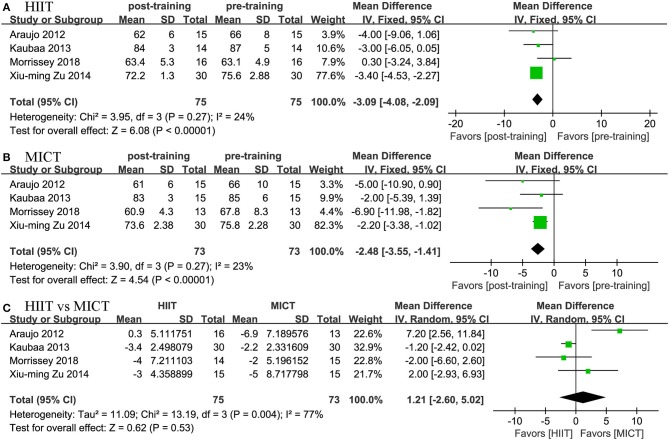
Forest plot for changes in DBP. **(A)** Within-group effects of HIIT. **(B)** Within-group effects of MICT. **(C)** Between-group effects of HIIT and MICT.

Four studies reported the effects of HIIT on SBP and DBP and compared them with the effects of MICT. We found positive effects of HIIT and MICT on blood pressure. In terms of SBP, we found a positive effect of HIIT (MD = −3.994 mmHg, 95% CI: −6.942 to −1.045, *p* = 0.003) and MICT (MD = −3.089 mmHg, 95% CI: −5.679 to −0.498, *p* = 0.019). In terms of DBP, we also found a positive effect of HIIT (MD = −3.087 mmHg, 95% CI: −4.083 to −2.092, *p* = 0.0001) and MICT (MD = −2.481 mmHg, 95% CI: −3.551 to −1.410, *p* = 0.0001). The two groups did not differ significantly; no significant difference was established by the meta-analysis for the change in blood pressure between the HIIT and MICT interventions (HIIT, MD = −1.208 mmHg, 95% CI: −2.603 to 0.186, *p* = 0.089; MICT, MD = 1.213 mmHg, 95% CI: −2.597 to 5.023, *p* = 0.533; Figures 6, 7).

#### Cardiorespiratory Fitness

**Figure 8:**
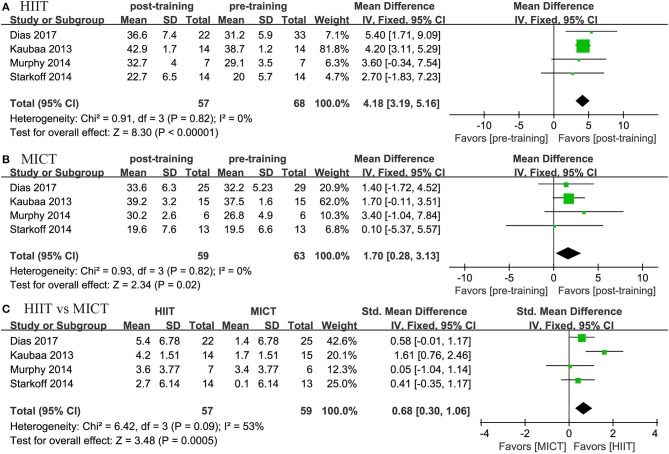
Forest plot for changes in VO_2peak_. **(A)** Within-group effects of HIIT. **(B)** Within-group effects of MICT on body weight. **(C)** Between-group effects of HIIT and MICT.

Four studies reported the effects of HIIT on VO_2peak_ and compared them with the effects of MICT. The meta-analysis showed that both HIIT and MICT could significantly improve VO_2peak_ (HIIT, MD = 4.17 mL/kg/min, 95% CI: 3.191 to 5.163, *p* = 0.0001; MICT, MD = 1.704 mL/kg/min, 95% CI: 0.279 to 3.130, *p* = 0.019). The pooled results of the meta-analysis also revealed that HIIT had a more positive effect on VO_2peak_ than MICT (MD = 2.497 mL/kg/min, 95% CI: 1.151 to 3.843, *p* = 0.0001; Figure 8).

### Subgroup Analysis

Subgroup analysis was conducted based on the HIIT training parameters of the training session time, duration, and interval protocol. The subgroup analysis results revealed that duration was a key parameter associated with cardiorespiratory fitness improvement. HIIT programs of ≥8 weeks showed positive effects on VO_2peak_ (SMD = 0.805, 95% CI: 0.334 to 1.276, *p* = 0.001) compared with MICT. However, HIIT programs of <8 weeks did not show any positive effects on VO_2peak_ (SMD = 0.276, 95% CI: −0.346 to 0.904, *p* = 0.381). Furthermore, long-interval HIIT programs seemed to more effectively improve VO_2peak_ (SMD = 0.691, 95% CI: 0.290 to 1.092, *p* = 0.006) than short-interval HIIT programs. HIIT also demonstrated positive effects on VO_2peak_ (SMD = 0.468, 95% CI: 0.040 to 0.897, *p* = 0.006) and LDL-c (SMD = −0.777, 95% CI: −1.456 to −0.098, *p* = 0.028), which were greater than the effects of MICT (Table 3).

**Table 3:** Subgroup analysis for the change in cardiometabolic risk factors in HIIT and MICT.

**Outcomes**		***N***	**ES (95% CI)**	**Heterogeneity**		***p***
				***I*^**2**^ (%)**	***P***	
Body weight	>8 weeks	5	SMD: −0.146 (−0.429, 0.138)	40.9	0.149	0.314
	≤8 weeks	2	SMD: −0.212 (−0.789, 0.365)	9.1	0.294	0.472
	Long interval	4	SMD: −0.373 (−0.753, 0.008)	44.7	0.164	0.055
	Short interval	3	SMD: 0.015 (−0.327, 0.357)	0	0.554	0.993
	Same time	3	SMD: −0.044 (−0.448, 0.361)	47.0	0.152	0.833
	Less time	3	SMD: −0.348 (−0.714, 0.018)	0	0.419	0.062
BMI	>8 weeks	5	SMD:0.273 (−0.012, 0.557)	0	0.518	0.061
	≤ 8 weeks	3	SMD:0.034 (−0.477, 0.545)	0	0.925	0.896
	Long interval	4	SMD:0.080 (−0.264, 0.425)	0	0.982	0.648
	Short interval	4	SMD:0.365 (−0.005, 0.725)	0	0.456	0.047
	Same time	3	SMD:0.349 (−0.075, 0.772)	21.1	0.282	0.106
	Less time	4	SMD:0.08 (−0.299, 0.46)	0	0.973	0.678
WC	>8 weeks	2	SMD: 0.2 (−0.435, 0.835)	34.1	0.218	0.538
	≤ 8weeks	1	SMD: −0.321 (−1.081, 0.439)	32.5	NA	0.408
	Long interval	2	SMD: 0.109 (−0.726, 0.945)	59.5	0.116	0.797
	Short interval	1	SMD: −0.117 (−0.833, 0.599)	NA	NA	0.749
	Same time	1	SMD: −0.321 (−1.081, 0.439)	NA	NA	0.408
	Less time	1	SMD: −0.117 (−0.833, 0.559)	NA	NA	0.749
Body fat	>8 weeks	3	SMD: −0.095 (−0.302, 0.492)	0	0.478	0.639
	≤ 8 weeks	2	SMD: −0.317 (−0.942, 0.309)	0	0.754	0.321
	Long interval	3	SMD: −0.115 (−0.517, 0.287)	0	0.897	0.575
	Short interval	2	SMD: 0.099 (−0.789, 0.988)	46.9	0.170	0.826
	Same time	3	SMD: −0.165 (−0.607, 0.276)	21.4	0.762	0.463
	Less time	2	SMD: 0.170 (−0.412, 0.752)	21.4	0.259	0.566
VO_2peak_	>8 weeks	2	SMD: 0.805 (0.334, 1.276)	0	0.345	0.001
	≤ 8 weeks	2	SMD:0.276 (−0.346, 0.904)	0	0.615	0.381
	Long interval	3	SMD: 0.691 (0.290, 1.092)	0	0.425	0.001
	Short interval	1	SMD: 0.50 (−1.041, 1.41)	NA	NA	0.928
	Same time	3	SMD: 0.468 (0.040, 0.897)	0	0.633	0.032
	less time	1	SMD: 1.109 (0.323, 1.895)	NA	NA	0.006
SBP	>8 weeks	4	SMD: −0.330 (−0.742, 0.082)	33.9	0.209	0.117
	≤ 8 weeks	0	NA	NA	NA	NA
	Long interval	1	SMD: 0.184 (−0.549, 0.918)	NA	NA	0.063
	Short interval	3	SMD: −0.330 (−0.742, 0.082)	11.8	0.322	0.024
	Same time	NA	NA	0	NA	NA
	Less time	3	SMD: −0.179 (−0.541, 0.183)	0	0.525	0.332
DBP	>8 weeks	4	MD: 0.128 (−0.589, 0.846)	77.5	0.004	0.726
	≤ 8 weeks	NA	NA	NA	NA	NA
	Long interval	3	SMD: 0.370 (−0.460, 1.20)	72.8	0.025	0.383
	Short interval	1	SMD: −0.497 (−1.011, 0.017)	NA	NA	0.058
	Same time	NA	NA	NA	NA	NA
	Less time	3	SMD: 0.286 (−0.679, 1.251)	83.9	0.002	0.561
TC	>8 weeks	5	SMD: 0.027 (−0.442, 0.496)	60.1	0.040	0.911
	≤ 8 weeks	1	SMD: −4.292 (−5.624, −2.959)	NA	NA	0.001
	Long interval	4	SMD: −0.802 (−2.387, 0.368)	31.2	0.228	0.545
	Short interval	2	SMD: −0.160 (−1.456, 0.337)	98.0	0.0001	0.430
	Same time	2	SMD: −2.314 (−6.097, 1.468)	96.2	0.0001	0.230
	Less time	3	SMD: 0.161 (−0.589, 0.912)	73.8	0.022	0.674
HDL–c	>8 weeks	4	SMD: −0.072 (−0.383, 0.240)	0	0.468	0.653
	≤ 8 weeks	1	SMD: 4.204 (2.891, 5.517)	NA	NA	0.001
	Long interval	3	SMD: 1.217(−0.888, 3.322)	94.8	0.0001	0.257
	Short interval	2	SMD: −0.067(−0.481, 0.346)	0	0.651	0.750
	Same time	2	SMD: 2.173 (−1.721, 6.066)	96.5	0.0001	0.274
	Less time	2	SMD: −0.067(−0.481, 0.346)	0	0.651	0.750
LDL–c	>8 weeks	4	SMD: −0.158 (−0.548, 0.232)	33.3	0.213	0.427
	≤ 8 weeks	1	SMD: −1.166 (−1.944, −0.388)	NA	NA	0.003
	Long interval	2	SMD: −0.042 (−0.921, 0.836)	0	0.627	0.925
	Short interval	3	SMD: −0.526 (−1.170, 0.117)	64.4	0.060	0.109
	Same time	2	SMD: −0.777 (−1.456, −0.098)	46.9	0.170	0.025
	Less time	2	SMD: −0.209 (−0.639, 0.222)	5.4	0.304	0.342
TG	>8 weeks	5	SMD: −0.092(−0.379, 0.194)	0	0.799	0.529
	≤ 8 weeks	1	SMD: −0.966 (−1.725, −0.207)	NA	NA	0.013
	Long interval	3	SMD: −0.258 (−0.746, 0.230)	47.6	0.126	0.301
	Short interval	4	SMD: −0.126 (−0.540, 0.288)	0	0.681	0.550
	Same time	2	SMD: −0.588 (−1.251, 0.074)	46.1	0.173	0.082
	Less time	3	SMD: −0.024 (−0.385, 0.336)	0	0.566	0.894
HOMA–IR	>8 weeks	4	SMD: −1.102(−2.715, 0.511)	94.9	0.0001	0.180
	≤ 8 weeks	0	NA	NA	NA	NA
	Long interval	2	SMD: −0.264 (−0.740, 0.212)	0	0.603	0.277
	Short interval	2	SMD: −1.948 (−5.782, 1.886)	97.8	0.0001	0.319
	Same time	1	SMD: −0.158 (−0.780, 0.463)	NA	NA	0.618
	Less time	3	SMD: −1.430 (−3.709, 0.849)	96.2	0.001	0.219
Fasting glucose	>8 weeks	4	SMD: −2.507 (−5.108, 0.094)	97.2	0.0001	0.059
	≤ 8 weeks	0	NA	NA	NA	NA
	Long interval	2	SMD: −1.467 (−4.265, 1.321)	95.1	0.0001	0.302
	Short interval	2	SMD: −3.633 (−11.013, 3.747)	98.8	0.0001	0.335
	Same time	1	MD: −0.079 (−0.693, 0.535)	NA	NA	0.800
	Less time	3	MD: −3.374 (−7.401, 0.653)	97.8	0.0001	0.101
Fasting insulin	>8 weeks	3	SMD: −0.694 (−1.816, 0.428)	87.7	0.0001	0.225
	≤ 8 weeks	0	NA	NA	NA	NA
	Long interval	2	SMD: −0.140 (−0.653, 0.373)	0	0.581	0.592
	Short interval	1	SMD: −1.746 (−2.344, −1.149)	NA	NA	0.001
	Same time	0	NA	NA	NA	NA
	Less time	3	SMD: −0.694 (−1.816, 0.428)	87.7	0.0001	0.225

## Discussion

This meta-analysis is the first study to systematically compare the effectiveness of HIIT and MICT on cardiometabolic risk factors and cardiorespiratory fitness in childhood obesity. The results showed that HIIIT and MICT were effective interventions in reducing cardiometabolic risk through body weight, BMI, SBP, and DBP reduction. However, no significant differences were noted in these effects in the comparison between the HIIT and MICT interventions. Furthermore, the meta-analysis revealed that the post-intervention change in VO_2peak_ was significantly greater following HIIT than following MICT at the same training session. With no significant adverse events reported, these findings indicated that HIIT is an appropriate and alternative training modality to MICT for reducing cardiometabolic risk in childhood obesity.

We found meaningful reductions from pre- to post-intervention of −5.45 and −4.604 kg in body weight and −1.661 and −2.366 kg/m^2^ in BMI in the HIIT and MICT groups, respectively, with no difference in the change in body weight or BMI in the pooled results and subgroup analysis. Therefore, our results confirmed that both HIIT and MICT improved body composition to a similar extent in childhood obesity. These findings were consistent with those reported by Keating et al. (2017), whose meta-analysis combined 31 studies involving 873 participants and demonstrated that both HIIT and MICT are equally beneficial for eliciting a small reduction in body fat when a similar time commitment or energy expenditure is used in young adults and adults who are overweight or obese. A recent meta-analysis conducted by Wewege et al. (2017) also found that HIIT and MICT induce a similar magnitude of change in body fat and WC in overweight and obese adults. Given the magnitude and statistics of the effect sizes associated with the training session time, duration, and interval protocol, subgroup analysis was conducted to explore whether different HIIT training parameters cause changes in body composition compared with MICT. The subgroup analysis demonstrated that none of the body composition measures in any subgroup elicited greater changes in HIIT than in MICT. These findings suggested that HIIT may be an effective alternative to MICT, achieving equivalent levels of body composition improvement. Moreover, although there was no difference in body composition improvement between the HIIT and MICT interventions, the physiological nature of HIIT and MICT differed. First, moderate-intensity exercise may involve elevated rates of burning of fat as a substrate, with a sustained high release of free fatty acids (FFAs) and subsequent oxidation of FFAs, whereas high-intensity exercise may be associated with the increased secretion of catecholamine and growth hormone, which could improve the rates of adipose lipolysis (Jensen, 2003; Trapp et al., 2007; van Hall, 2015). Second, high-intensity exercise elicits high excess post-exercise oxygen consumption, which promotes a substrate shift that favors fat utilization during the recovery period (Saris and Schrauwen, 2004; Islam et al., 2018). The findings of Treuth et al. (1996); Saris and Schrauwen (2004) supported this inference, demonstrating that high-intensity exercise and moderate-intensity exercise are similar for fat consumption within 24 h after exercise. These factors might explain why HIIT could achieve similar effects on body composition as MICT in obese children and adolescents.

Our studies indicated that both HIIT and MICT led to a small but significant reduction in SBP and DBP, which may have a positive effect on preventing hypertension in childhood obesity. The potential for blood pressure reduction is low, which may be related to the fact that our study did not include work based on hypertension. The absence of a significant difference in blood pressure reduction between HIIT and MICT was similar to the findings of previous systematic reviews and meta-analyses, indicating that HIIT and MICT could provide a similar decrease in SBP and DBP in adults with pre- to established hypertension (Costa et al., 2018). Campbell et al. (2019) also demonstrated that a HIIT-induced blood pressure reduction was comparable with that of MICT in adults; people who were overweight or obese were more responsive in terms of blood pressure reduction than people with a normal weight. All of these findings suggested that the effects of HIIT and MICT on blood pressure reduction were comparable; our study proved that those effects also exist in childhood obesity. The mechanism of HIIT in blood pressure reduction may be related to nitric oxide (NO). Previous research demonstrated that high-intensity exercise can increase the blood flow velocity, resulting in increased NO production by vascular endothelial cells, further vasodilation of blood vessels, and lowered blood pressure (Ghardashi Afousi et al., 2018; Izadi et al., 2018). Furthermore, HIIT could increase the shear stress of the vascular endothelium, reduce sympathetic nerve activity and peripheral blood vessel resistance, and lower blood pressure (Nishida et al., 1992; Halliwill, 2001; Green et al., 2004; Pal et al., 2013; Sawyer et al., 2016).

Unfortunately, our study failed to provide sufficient evidence to confirm the effects of HIIT and MICT on glucose metabolism and lipid metabolism, even if an improvement was observed in both interventions. These findings were inconsistent with the results of previous studies, which found that MICT and HIIT, when implemented in early life, are effective in lowering the relative weight of adipose tissue and improving glucose metabolism, thereby reverting or preventing metabolic alterations (Marcinko et al., 2015; de Lade et al., 2018). Recent studies have found that a large proportion of individuals do not respond (non-responders) or respond adversely to exercise in terms of glycemic control (Atkinson and Batterham, 2015; Bohm et al., 2016). Some variables, such as exercise dose and other phenomena like genetics and gut microbiota, have been identified as the causes of response heterogeneity. We attempted to use subgroups to analyze the effects of different HIIT training parameters on glucose metabolism and lipid metabolism, but no significant changes were found. Recent studies indicated that the dysbiosis of gut microbiota plays a critical role in response to exercise; moreover, HIIT-induced alterations in the gut microbiota are correlated closely with improvements in glucose homeostasis and insulin sensitivity, and participants who do not respond to HIIT are characterized by increased production of metabolically detrimental compounds (Bouter et al., 2017; Liu Y. et al., 2019). However, research on gut microbiota in obese adolescents remains limited. Therefore, the mechanism of the gut microbiota in improving glycemic and lipid metabolism in non-response children with obesity should be further explored by high-quality studies.

Notably, a significant improvement from pre- to post-intervention of 4.17 and 1.704 mL/kg/min for VO_2peak_ was found in both of the HIIT and MICT interventions, with greater improvement observed in the HIIT intervention than in MICT. Therefore, both HIIT and MICT could effectively improve cardiorespiratory fitness in obese children and adolescents. From a clinical perspective, the improvement of cardiorespiratory fitness in childhood is associated with enhanced cardiometabolic health in later life. Schmidt et al. (2016) showed that low cardiorespiratory fitness in childhood is a significant independent predictor of metabolic syndrome (MS) in early adulthood; obese children with low cardiorespiratory fitness who increase their relative fitness by adulthood present substantially reduced risk of MS compared with those who maintain low fitness. Moreover, Lahoz-Garcia et al. (2018) demonstrated that cardiorespiratory fitness is a partial mediator of the relationship of energy and macronutrient intake with obesity. Previous studies have revealed that cardiorespiratory fitness as a mediator could reduce the rate of cardiovascular disease-induced mortality by 15% for every one metabolic equivalent in adults. All of these findings suggest the importance of improving cardiorespiratory fitness in obese children and adolescents for cardiometabolic risk reduction. Our meta-analysis demonstrated that HIIT improved cardiorespiratory fitness compared with MICT for the first time in obese children and adolescents; these results were in accordance with previous systematic reviews that investigated the effect of HIIT vs. MICT on VO_2peak_ in patients with heart failure (Gomes Neto et al., 2018) and type 2 diabetes (Liu et al., 2019b). Although the effects of HIIT on cardiorespiratory fitness have been confirmed in a variety of individuals, findings on cardiorespiratory fitness improvement compared with MICT are inconsistent. A recent meta-analysis conducted by Gomes-Neto et al. (2017) showed that HIIT is superior to MICT on VO_2peak_ gain in patients with heart failure, whereas this advantage disappears in comparison with an isocaloric MICT protocol. Moreover, the meta-analysis of Milanovic et al. (2015) demonstrated that both HIIT and endurance training can elicit a large improvement in VO_2max_ in healthy adults aged 18–45 years and that HIIT has a small beneficial effect on VO_2max_ compared with endurance training. Given the magnitude and effect sizes associated with the training session time, duration, and interval protocol, we conducted a subgroup analysis to explore whether different HIIT training parameters cause changes in cardiometabolic health compared with those of MICT. Subgroup analysis indicated that the duration was a moderator for VO_2peak_, with larger effects evident in studies of ≥8 weeks compared with those with a <8-week duration. These findings were partially consistent with Hannan et al.'s meta-analysis, which found that patients with coronary artery disease who engaged in HIIT intervention for ≤6 weeks did not experience significant changes compared with MICT intervention, while 7–12 weeks may be a reasonable duration for the largest improvement in cardiorespiratory fitness (Hannan et al., 2018). The underlying physiological mechanisms that may explain why HIIT elicited a greater improvement than MICT are not fully understood. Several physiological adaptations may partially explain the potential mechanism, which involves central adaption and peripheral adaptation. HIIT-induced central adaptation primarily increased the ejection volume due to the increased pre-load, decreased afterload, and cardiac enlargement (Nottin et al., 2002). Meanwhile, peripheral adaptation may be related to skeletal muscle remodeling, which primarily improves capillary and mitochondrial density and increases skeletal muscle oxidative capacity, as reflected by the maximal activity and protein content of mitochondrial enzymes (Gibala et al., 2012; Montero et al., 2015; Lundby and Jacobs, 2016).

The current study is the first meta-analysis of RCTs to evaluate the difference between the effects of HIIT and of MICT on cardiometabolic risk factors among children and adolescents with obesity. To ensure the robustness of our results, we investigated whether several HIIT training parameters (including the training session time, total training duration, and length of interval) affect the final results/pooled results. However, this study had several limitations that possibly affected the interpretation of our results. First, although we searched the relevant studies as thoroughly as possible, the small number of available RCTs limited the number of studies in the subgroups and prevented further meta-regression analysis to investigate the dose-response relationship between HIIT and cardiometabolic risk improvement. Second, the difference in the measurements of glucose metabolism and lipid metabolism of the included studies possibly led to the high heterogeneity, which may partly explain the non-significant improvement in both the HIIT and MICT groups. Third, the puberty of subjects may be an important factor affecting the results. Given that only three of the included studies evaluated the pubertal growth of subjects, we were unable to further explore the impact of the puberty stage on the cardiometabolic risk improvement between HIIT and MICT. Fourth, most of the included studies lacked information on whether HIIT and MICT have the same workload and attendance rates, which may adversely affect our pooled results. Despite these limitations, this meta-analysis provided a comprehensive analysis of all of the included studies to compare the effects of HIIT vs. MICT on cardiometabolic risk and cardiorespiratory fitness in children and adolescents with obesity. Further studies with large samples and a high-quality methodology are needed to compare the effects of HIIT and MICT on glucose metabolism and lipid metabolism, determine the optimal HIIT protocol, and optimize the combination of training and intervals for maximum health benefits in children and adolescents with obesity.

In conclusion, the present meta-analysis supports the positive effects of HIIT on cardiorespiratory fitness and suggests that HIIT and MICT have similar effects on body composition and blood pressure in childhood obesity. These findings indicate that HIIT can be implemented in the management of childhood obesity as an alternative training modality to MICT to maintain cardiometabolic health.

## Author Contributions

JL contributed to the study conception and design, drafted the submitted article, and critically revised the draft for important intellectual content. LZ revised the draft for important intellectual content and gave the final approval of the version for publication. YS contributed to the acquisition, analysis, and interpretation of the data.

### Conflict of Interest

The authors declare that the research was conducted in the absence of any commercial or financial relationships that could be construed as a potential conflict of interest.
