# Supplementary material for: Comparative Effectiveness of High-Intensity Interval Training and Moderate-Intensity Continuous Training for Cardiometabolic Risk Factors and Cardiorespiratory Fitness in Childhood Obesity: A Meta-Analysis of Randomized Controlled Trials
Source: Front Physiol. 2020 Apr 3;11:214. doi: 10.3389/fphys.2020.00214 (PMC7145974; doi:10.3389/fphys.2020.00214)
Supplement: Supplementary file 1 [file Data_Sheet_1.pdf]

Supplementary appendix e-1: Search strategies for all databases.

1. Search Strategy for Pubmed: = 147

#1 "pediatric obesity" [MeSH Terms] OR "Obesity, Pediatric"[Title/Abstract] OR "Childhood Onset Obesity"[Title/Abstract] " OR "Obesity, Childhood Onset"[Title/Abstract] OR "Obesity in Childhood"[Title/Abstract] OR "Child Obesity"[Title/Abstract] OR "Obesity, Child"[Title/Abstract] OR "Childhood Obesity"[Title/Abstract] OR "Obesity, Childhood"[Title/Abstract] OR "Infant Overweight"[Title/Abstract] OR "Overweight, Infant"[Title/Abstract] OR "Infantile Obesity"[Title/Abstract] OR "Obesity, Infantile"[Title/Abstract] OR "Infant Obesity"[Title/Abstract] OR "Obesity, Infant"[Title/Abstract] OR "Childhood Overweight"[Title/Abstract] OR "Overweight, Childhood"[Title/Abstract] OR "Adolescent Overweight"[Title/Abstract] OR "Overweight, Adolescent"[Title/Abstract] OR "Adolescent Obesity"[Title/Abstract] OR "Obesity, Adolescent"[Title/Abstract] OR "Obesity in Adolescence"[Title/Abstract] OR "Obesity in Childhood"[Title/Abstract] OR "Child Obesity"[Title/Abstract] OR "Adolescent Obesity"[Title/Abstract] OR "Obesity, Pediatric"[Title/Abstract] OR "Obesity, Child"[Title/Abstract] OR "Childhood Obesity"[Title/Abstract] OR "Obesity in Adolescence"[Title/Abstract]

#2 "High Intensity Interval Training" [MeSH Terms]" OR "High-Intensity Interval Trainings"[Title/Abstract]" OR "Interval Training, High-Intensity"[Title/Abstract] OR "Interval Trainings, High-Intensity"[Title/Abstract] OR "Training, High-Intensity Interval"[Title/Abstract] OR "High-Intensity Intermittent Exercise"[Title/Abstract] OR "Exercise, High-Intensity Intermittent"[Title/Abstract] OR "Exercises, High-Intensity Intermittent"[Title/Abstract] OR "High-Intensity Intermittent Exercises"[Title/Abstract] OR "Sprint Interval Training"[Title/Abstract] OR "Sprint Interval Trainings"[Title/Abstract]

#3 "randomized controlled trial" [Publication Type] OR "controlled clinical trial" [Publication Type] OR "Single-Blind Method" [Text Word] OR "Double-Blind Method" [Text Word] OR "random allocation" [Text Word] OR "allocation" [Text Word] OR "RCT" [Text Word]

#4 #1 AND #2 AND #3

web of science=25

#1 TS= "pediatric obesity" OR TI= "Obesity, Pediatric OR Childhood Onset Obesity OR Obesity, Childhood Onset OR Obesity in Childhood OR Child Obesity OR Obesity, Child

OR Childhood Obesity OR Obesity, Childhood OR Infant Overweight OR Overweight, Infant OR Infantile Obesity OR Obesity, Infantile OR Infant Obesity OR Obesity, Infant OR Childhood Overweight OR Overweight, Childhood OR Adolescent Overweight OR Overweight, Adolescent OR Adolescent Obesity OR Obesity, Adolescent OR Obesity in Adolescence OR Obesity in Childhood OR Child Obesity OR Adolescent Obesity OR Obesity, Pediatric OR Obesity, Child OR Childhood Obesity OR Obesity in Adolescence”

#2 TS= “High Intensity Interval Training” OR TI= “High-Intensity Interval Trainings OR Interval Training, High-Intensity OR Interval Trainings, High-Intensity OR Training, High-Intensity Interval OR High-Intensity Intermittent Exercise OR Exercise, High-Intensity Intermittent OR Exercises, High-Intensity Intermittent OR High-Intensity Intermittent Exercises OR Sprint Interval Training OR Sprint Interval Trainings”

#3 TS= “Randomized controlled trial” OR TI= controlled clinical trial OR Single-Blind Method OR Double-Blind Method OR random allocation OR allocation OR RCT”

#4 #1 AND #2 AND #3

The Cochrane Library=55

#1 MeSH descriptor: [Pediatric Obesity] explode all trees

#2 (Obesity, Pediatric OR (pediatric obesity) OR (Obesity, Pediatric) OR (Childhood Onset Obesity) OR (Obesity, Childhood Onset) OR (Obesity in Childhood) OR (Child Obesity) OR (Obesity, Child) OR (Childhood Obesity) OR (Obesity, Childhood) OR (Infant Overweight) OR (Overweight, Infant) OR (Infantile Obesity) OR (Obesity, Infantile) OR (Infant Obesity) OR (Obesity, Infant) OR (Childhood Overweight) OR (Overweight, Childhood) OR (Adolescent Overweight) OR (Overweight, Adolescent) OR (Adolescent Obesity) OR (Obesity, Adolescent) OR (Obesity in Adolescence) OR (Obesity in Childhood) OR (Child Obesity) OR (Adolescent Obesity) OR (Obesity, Pediatric) OR (Obesity, Child) OR (Childhood Obesity) OR (Obesity in Adolescence):ti,ab,kw in Trials

#3 MeSH descriptor: [High-Intensity Interval Training] explode all trees

#4 ((High Intensity Interval Training) OR (High-Intensity Interval Trainings) OR (Interval Training, High-Intensity) OR (Interval Trainings, High-Intensity) OR (Training, High-Intensity Interval) OR (High-Intensity Intermittent Exercise) OR (Exercise, High-Intensity Intermittent) OR (Exercises, High-Intensity Intermittent) OR (High-Intensity Intermittent Exercises) OR (Sprint Interval Training) OR (Sprint Interval Trainings)):ti,ab,kw

#5 MeSH descriptor: [Randomized Controlled Trial] explode all trees

#6 ((random*) or allocation or (random allocation) or placebo or single blind or double blind or (randomized controlled trial*) or RCT or (clinical trial*)):ti,ab,kw

#7 #1 OR #2

#8 #3 OR #4

#9 #5 OR #6

#10 #7 AND #8 AND #9

EMBASE=25

#1 'childhood obesity'/de OR 'pediatric obesity':ab,ti OR 'childhood onset obesity':ab,ti OR 'obesity, childhood onset':ab,ti OR 'obesity, childhood':ab,ti OR 'infant overweight':ab,ti OR 'overweight, infant':ab,ti OR 'infantile obesity':ab,ti OR 'obesity, infantile':ab,ti OR 'infant obesity':ab,ti OR 'obesity, infant':ab,ti OR 'childhood overweight':ab,ti OR 'overweight, childhood':ab,ti OR 'adolescent overweight':ab,ti OR 'overweight, adolescent':ab,ti OR 'obesity, adolescent':ab,ti OR 'obesity in childhood':ab,ti OR 'child obesity':ab,ti OR 'adolescent obesity':ab,ti OR 'obesity, pediatric':ab,ti OR 'obesity, child':ab,ti OR 'childhood obesity':ab,ti OR 'obesity in adolescence':ab,ti

#2 "High Intensity Interval Training":ab,ti OR "High-Intensity Interval Trainings":ab,ti OR "Interval Training, High-Intensity":ab,ti OR "Interval Trainings, High-Intensity":ab,ti OR "Interval Trainings, High-Intensity":ab,ti OR "Interval Trainings, High-Intensity":ab,ti OR "Training, High-Intensity Interval":ab,ti OR "High-Intensity Intermittent Exercise":ab,ti OR "Exercise, High-Intensity Intermittent":ab,ti OR "Exercises, High-Intensity Intermittent":ab,ti OR "High-Intensity Intermittent Exercises":ab,ti OR "Sprint Interval Training":ab,ti OR "Sprint Interval Trainings":ab,ti OR “high intensity interval training”/exp

#3 #1 AND #2

EBSCO= 80

#1 (SU pediatric obesity）OR （TI/AB Obesity, Pediatric OR Childhood Onset Obesity OR Obesity, Childhood Onset OR Obesity in Childhood OR Child Obesity OR Obesity, Child OR Childhood Obesity OR Obesity, Childhood OR Infant Overweight OR Overweight, Infant OR Infantile Obesity OR Obesity, Infantile OR Infant Obesity OR Obesity, Infant OR Childhood Overweight OR Overweight, Childhood OR Adolescent Overweight OR Overweight, Adolescent OR Adolescent Obesity OR Obesity, Adolescent OR Obesity in Adolescence OR Obesity in Childhood OR Child Obesity OR Adolescent Obesity OR Obesity, Pediatric OR Obesity, Child OR Childhood Obesity OR Obesity in Adolescence ）

2 (SU high intensity interval training) OR (TI/AB High-Intensity Interval Trainings OR Interval Training, High-Intensity OR Interval Trainings, High-Intensity OR Training, High-Intensity Interval OR High-Intensity Intermittent Exercise OR Exercise, High-Intensity Intermittent OR Exercises, High-Intensity Intermittent OR High-Intensity Intermittent Exercises OR Sprint Interval Training OR Sprint Interval Trainings)

3 #1 AND #2

CNKI = 68

#1 SU=青少年肥胖 OR TI=肥胖儿童 OR TI=肥胖青少年 OR TI=儿童肥胖 OR AB=肥胖

#2 SU=高强度间歇训练 OR TI=高强度间歇 OR TI=间歇运动 OR TI=高强度运动 OR TI=高强度有氧运动 OR TI=有氧间歇运动 OR TI=高强度间歇运动

#3 #1 AND #2
